# Supplementary material for: An ensemble approach for research article classification: a case study in artificial intelligence
Source: PeerJ Comput Sci. 2024 Dec 10;10:e2521. doi: 10.7717/peerj-cs.2521 (PMC11784741; doi:10.7717/peerj-cs.2521)
Supplement: Supplemental Information 1 [file peerj-cs-10-2521-s001.docx]

Lexical query in Tang's approach

TS=("action recognition" OR "activation function\$" OR "activity recognition" OR "adaboost" OR "AI" OR "algorithm\$" OR "anfis" OR "ann" OR "anomaly detection" OR "ant colony optimization" OR "artificial bee colony" OR "artificial neural-network\$" OR "artificial-intelligence" OR "attribute reduction" OR "augmented reality" OR "autoencoder\$" OR "automa* detection" OR "automa* segmentation" OR "automa* classification" OR "background subtraction" OR "backpropagation" OR "bankruptcy prediction" OR "bayesian network\$" OR "bayesian-inference" OR "bidirectional lstm" OR "big data" OR "bootstrap" OR ("brain-computer interface\$" OR "brain-computer-interface\$") OR "canonical correlation-analysis" OR "cellular neural-network\$" OR "classifier\$" OR ("cluster-analysis" OR "cluster analysis") OR "cnn" OR "community detection" OR "complex dynamical network\$" OR "component analysis" OR "computational intelligen*" OR "computer vision" OR "computer-aided detection" OR "concept drift" OR "consensus model" OR ("convolutional network\$" OR "convolutional neural-network\$") OR "corpus" OR "crack detection" OR "cross-validation" OR "damage detection" OR "data augmentation" OR "data fusion" OR "data mining" OR "decision tree\$" OR "deconvolution" OR "deep neural-network\$" OR "defect detection" OR "dempster-shafer theory" OR "differential evolution" OR "dimensionality reduction" OR "discriminant-analysis" OR "dynamical network\$" OR "edge-detection" OR "eigenface\$" OR "emotion recognition" OR "energy minimization" OR "event detection" OR "evidential belief function" OR "evidential reasoning approach" OR "expert-system" OR "exponential stability" OR "exponential synchronization" OR "expression recognition" OR "extended kalman filter" OR "extreme learning-machine\$" OR ("face recognition" OR "face-recognition") OR "facial expression recognition" OR "fault-diagnosis" OR "feature subset-selection" OR "feature-extraction" OR "feature-selection" OR "feedforward networks" OR "fuzzy c-means" OR "fuzzy inference system" OR "fuzzy-logic" OR "fuzzy-set\$" OR "fuzzy-system\$" OR "gan" OR "gaussian process regression" OR "generative adversarial network\$" OR "gesture recognition" OR "global exponential stability" OR "gradient descent" OR "grey wolf optimizer" OR "group decision-making" OR "hidden markov-models" OR "human activity recognition" OR "image classification" OR "image registration" OR "image segmentation" OR "image-analysis" OR "imbalanced data" OR "inference system" OR "information extraction" OR "in-silico prediction" OR "intrusion detection" OR "kalman filter" OR "kernel" OR "k-svd" OR "lasso" OR "lda" OR "leader-following consensus" OR "learning framework" OR "learning-based optimization" OR "learning-model" OR "least-squares" OR "linear discriminant-analysis" OR "local binary patterns" OR "logistic-regression" OR "lstm" OR "machine vision" OR "markovian jump systems" OR "metaheuristics" OR "multiagent system\$" OR "multilayer feedforward network\$" OR "multilayer perceptron" OR "multiobjective optimization" OR "naive bayes" OR "nearest-neighbor" OR "neural-control" OR "neural-network\$" OR "nonlinear dimensionality reduction" OR "nonlinear-systems" OR "novelty detection" OR ("object detection" OR "object recognition") OR "object tracking" OR "outlier detection" OR "parameter-estimation" OR "parameter-identification" OR "partial least-squares" OR "particle swarm" OR "pattern-classification" OR "pattern-recognition" OR "pca" OR "pcnn" OR "pedestrian detection" OR "perceptron" OR "permutation entropy" OR "person reidentification" OR "pls" OR "pose estimation" OR "principal component analysis" OR "pso" OR "quantile regression" OR "random forest\$" OR "recommender system\$" OR "recurrent neural-network\$" OR "regression-analysis" OR "regression-models" OR "representation model" OR "robot" OR "robot manipulator\$" OR "roc curve" OR "rough set\$" OR "rule extraction" OR "scene classification" OR "seizure detection" OR "self-organizing map\$" OR "semantic segmentation" OR "semantic similarity" OR "semantic web" OR "sentiment analysis" OR "sequence-based predictor" OR "short-term-memory" OR "smote" OR "sparse representation" OR "species distribution model\$" OR ("support vector machine\$" OR "support-vector-machine") OR "support vector regression" OR "svm" OR "svr" OR "target detection" OR "text classification" OR "texture analysis" OR "texture classification" OR "time-series" OR "time-varying delay\$" OR "traffic flow prediction" OR "trajectory tracking" OR "travel-time prediction" OR "variable selection" OR "variational mode decomposition" OR "visual tracking" OR "visual-attention")
